# Molecular characterization of the duck enteritis virus US10 protein

**DOI:** 10.1186/s12985-017-0841-2

**Published:** 2017-09-20

**Authors:** Daixi Zhang, Maoyin Lai, Anchun Cheng, Mingshu Wang, Ying Wu, Qiao Yang, Mafeng Liu, Dekang Zhu, Renyong Jia, Shun Chen, Kunfeng Sun, Xinxin Zhao, Xiaoyue Chen

**Affiliations:** 1 Institute of Preventive Veterinary Medicine, Sichuan Agricultural University, Wenjiang, 611130 People’s Republic of China; 2 Avian Disease Research Center, College of Veterinary Medicine of Sichuan Agricultural University, Wenjiang, 611130 People’s Republic of China; 3 Key Laboratory of Animal Disease and Human Health of Sichuan Province, Sichuan Agricultural University, Wenjiang, 611130 People’s Republic of China

**Keywords:** Duck enteritis virus, US10, Kinetic class, True late gene, γ2 Gene, Subcellular localization, Virion protein

## Abstract

**Background:**

There is little information regarding the duck enteritis virus (DEV) US10 gene and its molecular characterization.

**Methods:**

Duck enteritis virus US10 was amplified and cloned into the recombinant vector pET32a(+). The recombinant US10 protein was expressed in *Escherichia coli* BL21 cells and used to immunize rabbits for the preparation of polyclonal antibodies. The harvested rabbit antiserum against DEV US10 was detected and analyzed by agar immunodiffusion. Using this antibody, western blotting and indirect immunofluorescence analysis were used to analyze the expression level and subcellular localization of US10 in infected cells at different time points. Quantitative reverse-transcription PCR (qRT-PCR) and pharmacological inhibition tests were used to ascertain the kinetic class of the US10 gene. A mass spectrometry-based strategy was used to identify US10 in purified DEV virions and quantify its abundance.

**Results:**

The recombinant pET32a(+)/US10 protein was expressed as inclusion bodies, purified by gradient urea washing, and used to prepare specific antibodies. The results of qRT-PCR, western blotting, and pharmacological inhibition tests revealed that US10 is mainly transcribed in the late stage of viral replication. However, the presence of the DNA polymerase inhibitor ganciclovir and the protein synthesis inhibitor cycloheximide blocked transcription. Therefore, US10 is a γ2 (true late) gene. Indirect immunofluorescence analysis showed that US10 proteins were initially diffusely distributed throughout the cytoplasm, but with the passage of time, they gradually relocated to a perinuclear region. The US10 protein was detected in purified DEV virions by mass spectrometry, but was not detected by western blotting, indicating that DEV US10 is a minor virion protein.

**Conclusions:**

The DEV US10 gene is a γ2 gene and the US10 protein is localized in the perinuclear region. DEV US10 is a virion component.

## Background

Duck viral enteritis (DVE) is an acute, hemorrhagic, highly contagious disease of waterfowl caused by duck enteritis virus (DEV) [1, 2]. Since it was first reported in the Netherlands, this fatal pathogen has resulted in significant economic losses in domestic and wild waterfowl due to high mortality and decreased egg production [3]. In 2015, DEV was classified as an *Alphaherpesvirinae* (genus *Mardivirus)* according to the 10th report of the International Committee on Taxonomy of Viruses (ICTV) [4].

The complete sequences of different DEV strains have been published in GenBank, including three field (virulent) strains (CHv, 2085, and CSC) and five attenuated strains (C-KCE, VAC, Clone-03, CV, and K) [5–9]. The smallest DEV US10 protein has been reported to be 168 aa (C-KCE), while the largest one has been found in the CHv strain (322 aa). The US10 sequence of VAC has 100% identity to those of Clone-03 and K, while the 2085 sequence is 100% identical to those of CHv and CSC. An absence of a thymidine at position 787 of CHv and 2085 strains result in modification of the downstream 35 amino acids and an additional stretch of 11 amino acids. This mutation is identical in virulent and absent in attenuated strains, suggesting that DEV US10 may be involved in the process of attenuation [6, 7].

DEV US10 homologs were found in MDV, HVT, HSV-1, HSV-2, CeHV-1, EHV-1, EHV-4, VZV, and ILTV. The percentage homology ranges from 18.3 to 31.0% [8, 9]. The US10 homologs of EHV-1, HSV-1, and VZV are known to possess a sequence of 13 amino acids (C-X3-C-X3-H-X3-C), which is a perfect match to the consensus CCHC-type zinc finger domain [8–11]. The US10 gene is predicted to encode a tegument phosphoprotein in other alphaherpesviruses that can interact with host proteins and other viral proteins to play a role in virulence and pathogenicity [10, 12, 13]. However, the molecular characteristics and related functions of the DEV US10 gene have not yet been reported. Therefore, we expressed the recombinant US10 protein in a prokaryotic expression system to generate antiserum that recognizes US10 to better investigate its expression levels and subcellular localization in DEV-infected cells. The transcription phases and gene type of US10 were also determined through RT-PCR and pharmacological inhibition tests. We also detected US10 protein and its relative abundance in extracellular DEV virions by western blotting and mass spectrometry. This work provides a foundation for further studies on the function of DEV US10.

## Methods

### Viruses, strains, vectors, and other significant materials

The CHv strain of DEV (GenBank accession number: JQ647509), *E. coli* BL21 cells, *E. coli* DH5α cells, the prokaryotic expression vector pET-32a(+), pMD18-T/β-actin, the rabbit anti-DEV serum, and rabbit anti-gC serum were preserved and provided by the Avian Diseases Research Center, College of Veterinary Medicine, Sichuan Agricultural University. The pET32a(+)/US10 vector was constructed by Takara Biotechnology Co. (Dalian, China). Monolayer cultures of duck embryo fibroblasts (DEFs) were cultured in Minimal Essential Medium (MEM; Gibco) containing 10% fetal bovine serum (FBS; Gibco) and 100 μg/mL streptomycin. After DEV inoculation, the DEFs were incubated in MEM containing 3% FBS.

### PCR amplification and plasmid construction

All primers (Table 1) were designed by Oligo7.0, and P1/P2 were used to amplify DEV US10 (GenBank accession number: EU195084). The amplified product was sent to Takara to generate the prokaryotic expression plasmid pET32a(+)/US10 (data not shown). P3/P4 and P5/P6 were used to amplify DEV US10 and the duck β-actin gene by qRT-PCR, respectively. P7/P8 were used to amplify the DEV UL55 (GenBank: EU071034) gene, as a γ2 gene control [14, 15].

**Table 1:** Sequence and characteristics of primer pairs

Primer	Primer sequence (5′-3′)	Gene	Product size (bp)
P1	GAATTCATGAAGAGGCGCTGTCTCAAT	DEV US10	988
P2	AAGCTTTAGAGTATCAGTCAGAGTCATCGTAG		
P3	CATCCAGTTGCTCCCGT	DEV US10 (for qRT-PCR)	131
P4	GCGTGACCTAGACAACACC		
P5	CGGGCATCGCTGACA	Duck β-actin gene	177
P6	GGATTCATCATACTCCTGCTTGCT		
P7	AAGATGCTATGCTGCTAATA	DEV UL55	740
P8	CTGTTCGATCTTTACTATTA		

### Prokaryotic expression

The recombinant plasmid pET-32a(+)/US10 was transformed into *E. coli* BL21 cells, which were then induced using IPTG at a working concentration of 0.6 mM for 6 h at 37 °C. After centrifugation, the cells were resuspended in 20 mM Tris-HCl. The expression of the recombinant protein was determined using SDS-PAGE after the disruption of cells by cold sonication. The reactivity of recombinant proteins was determined by western blot analysis using rabbit anti-DEV serum as the primary antibody and HRP-labeled goat anti-rabbit IgG as the secondary antibody.

### Preparation and identification of the polyclonal antibody

The recombinant US10 protein was mainly expressed as inclusion bodies, purified through urea washing, dialysis, and renaturation, as described by Wu et al. [16]. Six healthy male rabbits were selected and immunized once a week to prepare the polyclonal antibody [17]. After four immunizations, blood was collected from the ear vein and the antibody titer was measured by the agar dilution method. After reaching the target titer, blood was taken from the heart to obtain rabbit anti-US10 polyclonal antibodies.

The reactivity of the antibody was determined using western blot analysis [18]. On the one hand, the lysates of bacteria transformed with pET-32a(+) vs. pET-32a(+)/US10 were blotted to test the cross-reactivity of anti-US10 serum. On the other hand, the DEV-infected or mock-infected DEFs were collected and probed with anti-US10 serum to detect the DEV US10 protein. HRP-labeled goat anti-rabbit IgG was used as the secondary antibody in two western blotting analyses.

### Real-time PCR (RT-PCR)

Total RNA was extracted from DEV-infected DEFs at different time points post-infection (0.5, 1, 2, 4, 6, 8, 12, 16, 24, 36, and 72 h) using Trizol, followed by DNase treatment during RNA extraction. The quality of the RNA samples was assessed and the samples were reverse-transcribed to cDNA, as described by Li et al. [19]. Subsequently, real-time PCR was performed in a 20-μL reaction volume containing 10 μL of SYBR Green Super Mix, 1 μL of each primer, 1 μL of cDNA, and 7 μL of ultrapure water. The thermal cycling procedure was carried out as follows: initial denaturation for 1 min at 95 °C followed by 45 cycles of denaturation at 95 °C for 5 s, annealing at 59 °C for 20 s, and extension at 72 °C for 25 s. Triplicate experiments were performed to analyze the expression of the US10 and β-actin genes, and the relative transcription level of the DEV US10 gene was calculated using the 2^−ΔCt^ method simplified from the 2^−ΔΔCt^ method. To evaluate the efficiency of each assay, standard curves were constructed using tenfold serial dilutions of pMD18-T/US10 and pMD18-T/β-actin.

### Pharmacological inhibition tests

Pharmacological inhibition tests were performed to confirm the kinetics of DEV US10 [10, 14]. Three bottles of DEFs were prepared and inoculated with DEV, one bottle without any drugs, and the other bottles containing 300 μg/mL ganciclovir (GCV, DNA polymerase synthesis inhibitor) or 100 μg/mL cyclohexamide (CHX, protein synthesis inhibitor). The infected cells were harvested 24 h after infection and washed twice with PBS. Extraction of total RNA and preparation of cDNA were performed similar to that described above for RT-PCR. The gene type of US10 was identified by PCR (UL55 and β-actin were used as a γ2 gene control and a housekeeping gene control, respectively).

### Indirect immunofluorescence assay (IFA)

IFA was conducted using a standard procedure [18]. Briefly, DEV-infected cells were harvested at 12, 24, 36, and 48 h post-infection, plated onto coverslips, and fixed with 4% paraformaldehyde for 30 min. The fixed cells were permeabilized with 0.5% Triton X-100 and incubated for 30 min in 5% BSA at 37 °C. The anti-US10 antibody and FITC-conjugated goat anti-rabbit IgG were used as primary and secondary antibodies, respectively, to sequentially probe the blots for 1 h. Subsequently, the cells were treated with DAPI for 10 min to stain the nucleus. Images were captured using a fluorescence microscope after the coverslips had been sealed with glycerin buffer on glass slides.

### Virion purification

DEF cells were mock-treated or infected with DEV CHv strain at an MOI of 5. At 2 hpi, the cells were washed twice with PBS and the medium was replaced by serum-free MEM. At 72 hpi, the medium was collected and clarified by low-speed centrifugation (2000×*g*, 20 min, 4 °C) to remove the cell debris. Extracellular DEV virions were harvested by ultracentrifugation (40,000×*g*, 2 h, 4 °C) through a 30% (wt/vol) sucrose cushion. Virions were then banded by isopycnic gradient ultracentrifugation in a continuous 30 to 60% (wt/vol) potassium tartrate gradient in TBS (40,000×*g*, 2 h, 4 °C). The band containing virions was collected, diluted tenfold in TBS, and pelleted by ultracentrifugation (20,000×*g*, 30 min, 4 °C). The pellet was finally resuspended in TBS and stored at −80 °C.

### Western blotting

Purified virion samples were boiled for 10 min in 5 × SDS-loading buffer and separated on 12% gels by SDS-PAGE. Proteins were transferred from the gels to polyvinylidene difluoride (PVDF) membranes, which were then blocked for 2 h in 5% BSA. All primary antibodies were diluted in blocking buffer and added to blots for 2 h. Blots were then washed three times in TBST and probed with HRP-conjugated goat anti-rabbit IgG secondary antibody.

### Mass spectrometry

Purified virion samples were separated by SDS-PAGE. The whole gel was stained with Coomassie brilliant blue and then sent to Sangon Biotech Company (Shanghai, China) for liquid chromatography–tandem mass spectrometry (LC-MS/MS) analysis. Details of in-gel trypsin digestion, LC-MS/MS, and database searches were as described previously by Loret et al. [20], Leroy et al. [21], and Johannsen et al. [22].

## Results

### Expression, identification, and purification of recombinant DEV US10

**Fig. 1:**
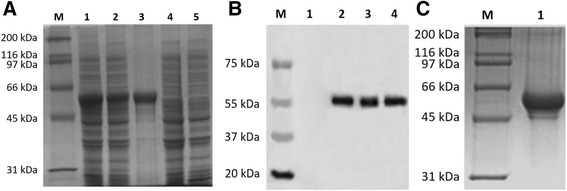
Expression, identification, and purification of the recombinant US10 protein. **a** Expression of the recombinant protein. Total protein stain. Lane M: markers; lanes 1–3: the whole bacterial lysate, supernatant, and inclusion bodies of pET32a(+)/US10; lanes 4 and 5: the induced and uninduced pET32a(+). **b** Identification of the recombinant protein by western blotting with anti-DEV serum; lane M: markers; lane 1: pET32a(+); lanes 2–4: the whole bacterial lysate, supernatant, and inclusion bodies of pET32a(+)/US10. **c** Purification of the recombinant US10 protein. Detection with anti-DEV serum. Lane M: markers; lane 1: purified recombinant pET32a(+)/US10 protein

The expression of recombinant protein (approximately 56.0 kDa, including DEV US10, a His-tag, and thioredoxin) was observed by SDS-PAGE after the plasmids pET-32a(+)/US10 and pET-32a(+) had been transformed into *E. coli* BL21 cells and induced by IPTG (Fig. 1a). Lanes 1–3 yielded target bands of 56 kDa, while no desired bands were observed in lanes 4 and 5, suggesting that the recombinant proteins were mainly expressed as inclusion bodies. Western blotting was then carried out to verify the reactivity of the recombinant protein. Rabbit anti-DEV serum and HRP-labeled goat anti-rabbit IgG were used as the primary and secondary antibodies, respectively. The results showed that the recombinant protein had good reactivity with the anti-DEV serum (Fig. 1b). Finally, the inclusion bodies were purified through urea washing (Fig. 1c) and used to immunize rabbits.

### Purification and verification of US10 antibodies

**Fig. 2:**
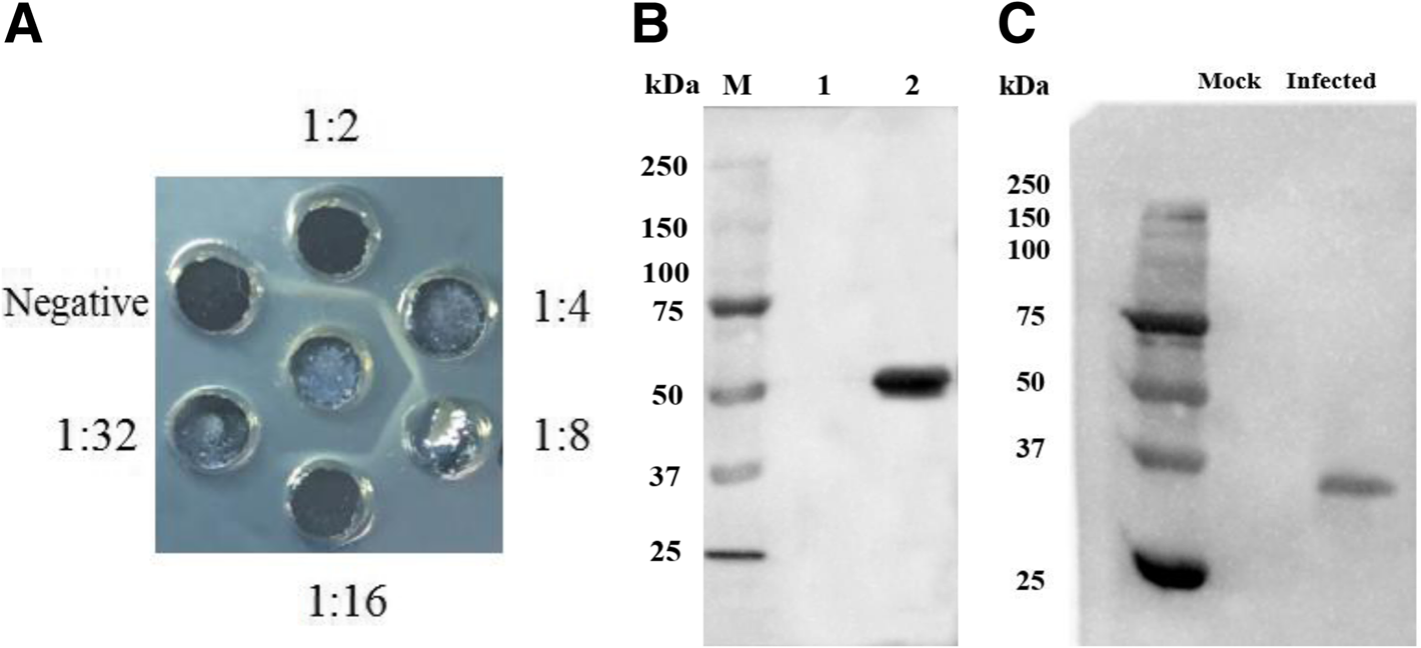
Preparation and verification of the polyclonal antibody raised against DEV US10. **a** Agar diffusion reaction test. Middle well: purified recombinant pET32a(+)/US10 protein. **b** Cross-reactivity test; lane M: markers; lane 1: the cell lysates of pET32a(+); lane 2: the cell lysates of pET32a(+)/US10. **c** The DEV US10 was recognized by purified polyclonal antibody. Lane 1: mock-treated DEFs; lane 2: DEV-infected DEFs

The harvested antiserum was titered by the agar diffusion assay. The results showed a maximum titer of 1:16 (Fig. 2a). The cell lysates of bacteria transformed with pET-32a(+) or pET-32a(+)/US10 were blotted with anti-US10 serum, and a specific band corresponding to a fusion protein of 56 kDa was obtained from pET-32a(+)/US10. This result indicated that the antibody is specific for DEV US10 and not cross-reactive with pET-32a(+) N-terminal vector-specific fusion elements (Fig. 2b). DEFs mock-infected or infected with DEV were also probed with anti-US10 serum to verify the ability to detect the desired protein. A target band of 36 kDa was obtained from infected DEFs, which is consistent with the expected size of DEV US10 (Fig. 2c). Thus, the rabbit anti-US10 serum can specifically recognize this protein.

### Kinetics of DEV US10

**Fig. 3:**
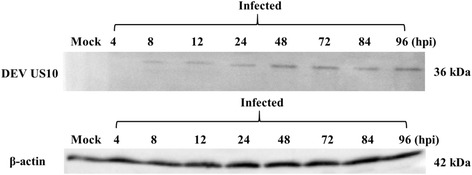
Expression of US10 protein and β-actin in DEV-infected cells. Proteins isolated from mock or DEV-infected cells at different times were subjected to western blot analysis with US10 or β-actin antiserum

Western blot analysis was performed using protein samples collected at 4, 8, 24, 48, 60, 72, 84, and 96 h post-infection (hpi). A specific protein band of approximately 36 kDa was first detected at 8 hpi; expression gradually increased and peaked at 72 hpi, but began to decline at 84 hpi. US10 was still detected at 96 hpi (Fig. 3).

**Fig. 4:**
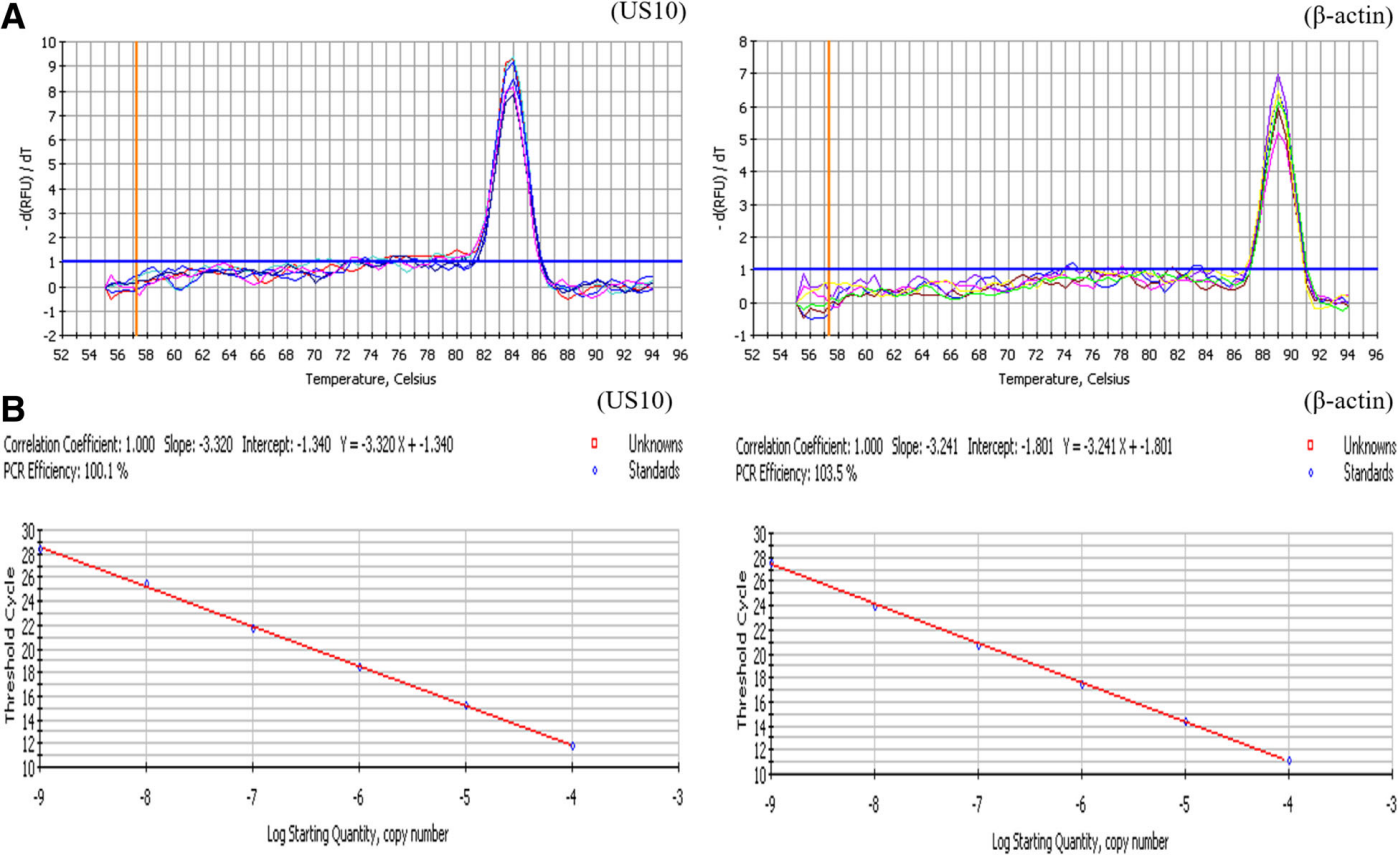
The melting curves and standard curves of DEV US10 gene and β-actin by qRT-PCR. **a**: The melting curve of the DEV US10 gene displayed a single peak at 84.0 °C, while β-actin displayed a single peak at 89.0 °C. **b**: The standard curves of DEV US10 and β-actin genes were calculated by iCycler IQ 5 software. Each dot represents the result of triplicate amplification of each dilution. The correlation coefficient and the slope of the regression curve were calculated and indicated. The standard curve equation of the DEV US10 gene is Y = −3.320X − 1.340, while that of the β-actin gene is Y = −3.241X − 1.801

**Fig. 5:**
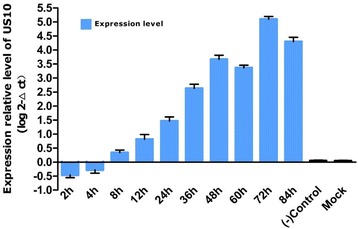
Transcriptional analysis of the DEV US10 gene. Total RNA was isolated from the DEV-infected DEF cells at each post-infection time point and converted to cDNA. Samples of cDNA were amplified using qPCR and SYBR green detection. Data are presented as the fold change in the expression of the DEV US10 gene. The transcriptional expression of the DEV US10 gene was normalized to that of a reference gene (β-actin)

We then investigated the relative expression of the DEV US10 gene in infected DEFs at different time points using qRT-PCR. The specificity of the primer sets was verified using a melting curve (Fig. 4a). Standard curves, plotting plasmid copy number against the Ct values for US10 (Y = −3.320X − 1.340) and β-actin (Y = −3.241X − 1.801), were established to evaluate the efficiency of the assays. The approximately identical amplification efficiencies of US10 (100.1%) and β-actin (103.5%) yielded a correlation coefficient of 1.00 (Fig. 4b). Subsequently, total RNA was isolated and reverse-transcribed to cDNA, followed by qPCR and data processing to analyze the US10 transcription level (Fig. 5). The assays showed that transcription began at 8 h and gradually increased, reaching a peak at 72 h. The expression and transcription levels of DEV US10 correlate with those of a γ (late) gene.

**Fig. 6:**
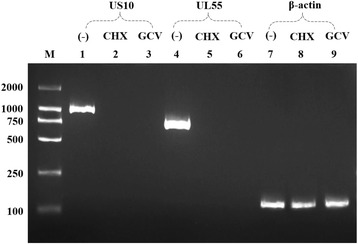
Pharmacological inhibition test showed that US10 is a true late gene. Lane M: markers; lanes 1, 4, and 7: DEV-infected cells, without any drugs; lanes 2, 5, and 8: DEV-infected cells treated with 100 μg/mL cyclohexamide (protein synthesis inhibitor); lanes 3, 6, and 9: DEV-infected cells treated with 300 μg/mL ganciclovir (DNA polymerase synthesis inhibitor). DEV UL55 and β-actin genes were used as a γ2 gene and a housekeeping gene, respectively

To further verify the US10 gene type, the DNA polymerase inhibitor GCV and the protein synthesis inhibitor CHX were used to determine kinetic class. As shown in Fig. 6, β-actin (amplified length of 177 bp) was detected regardless of the presence or absence of drugs, while US10 (amplified length of 988 bp) and UL55 (amplified length of 740 bp, DEV γ2 gene) were only detected when no drugs were present. Thus, US10 is a γ2 (true late) gene due to its strict dependence on the early synthesis of viral DNA and protein.

### Subcellular localization of DEV US10

**Fig. 7:**
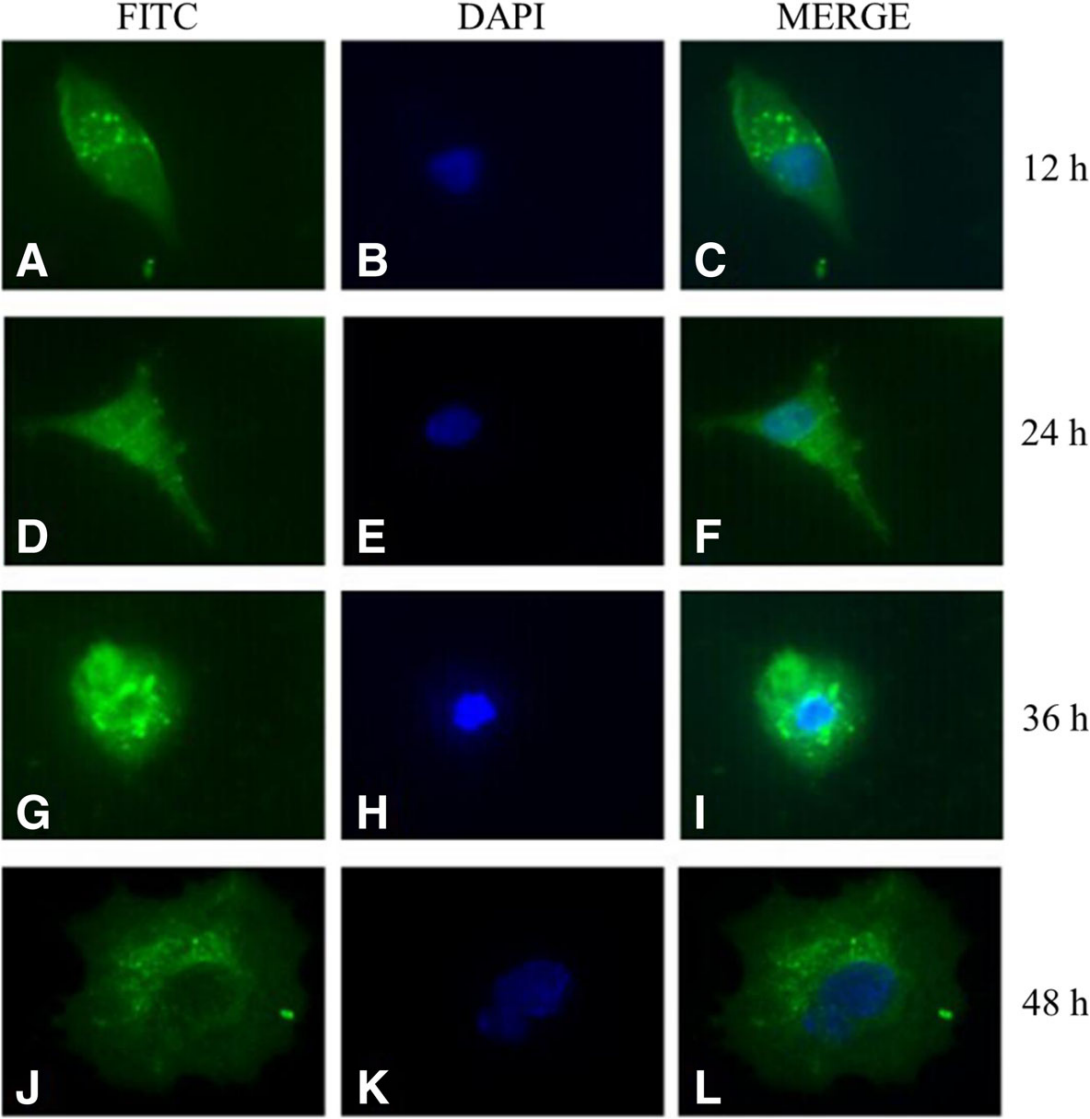
Subcellular localization of DEV US10 (400×). DEF cells were infected with DEV for 12, 24, 36, and 48 h. The cells were fixed, permeabilized, and stained with anti-US10 serum and FITC-conjugated goat anti-rabbit antibody, followed by DAPI. Panels **a**, **d**, **g**, **j**: US10 protein expressed in DEV-infected DEF cells. Panels **b**, **e**, **h**, **k**: nucleus of DEV-infected DEF cells. Panels **c**, **f**, **i**, **l**: US10 protein is localized in the perinuclear region

**Fig. 8:**
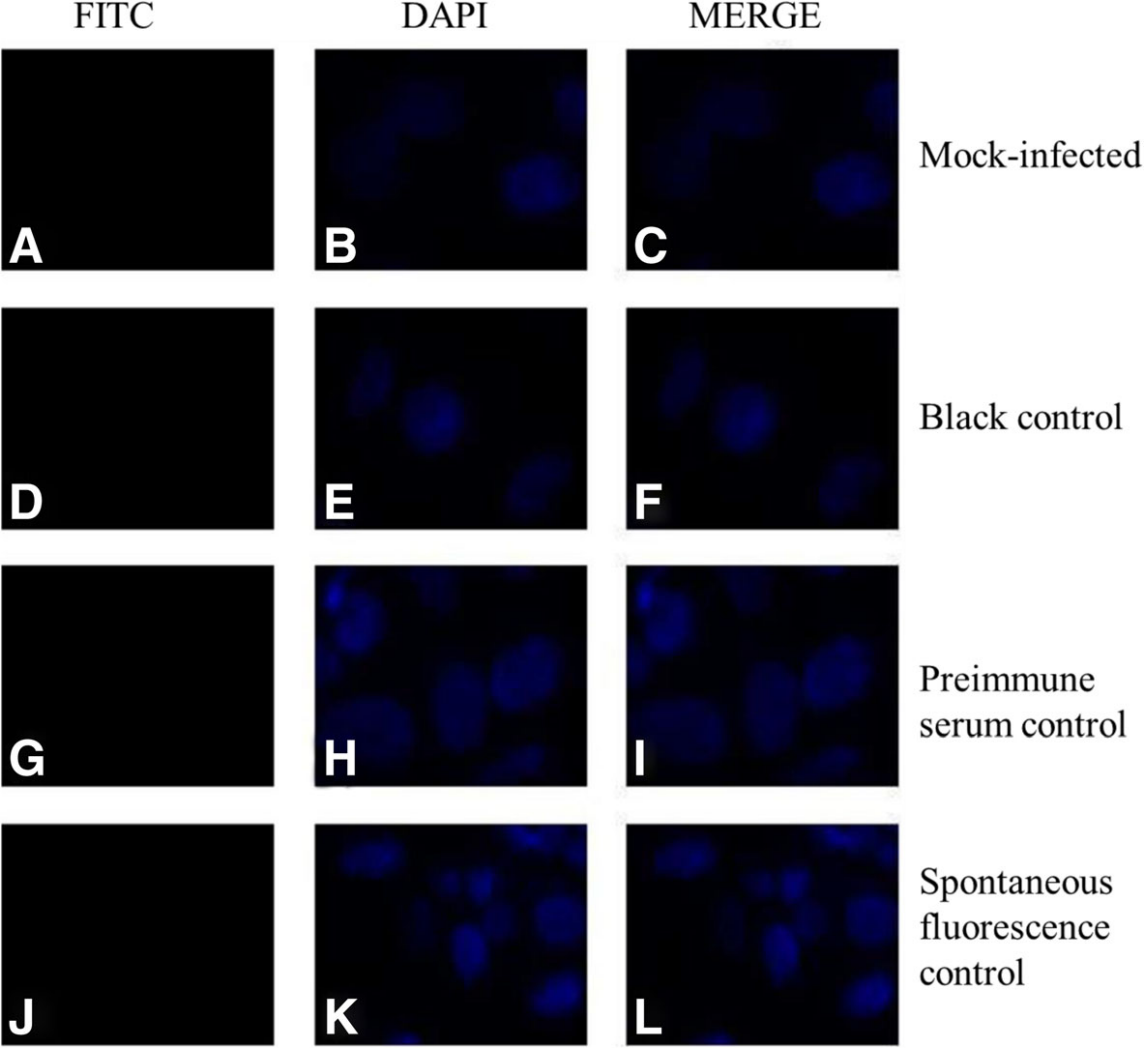
Mock-infected cells, blank control, preimmune serum control, and spontaneous fluorescence control subjected to immunofluorescence analysis (400×). Panels **a**, **d**, **g**, **j**: US10 protein was not detected in aboved control cells. Panels **b**, **e**, **h**, **k**: nucleus of aboved control cells. Panels **c**, **f**, **i**, **l**: There is no green fluorescence produced by false positives

The intracellular distribution of DEV US10 was confirmed through IFA using rabbit anti-US10 serum. US10 protein-specific fluorescence was primarily localized to the cytoplasm at 12 hpi, but then gradually relocated to a perinuclear region. At 48 hpi, almost all US10-specific fluorescence localized around the nucleus; subsequently, this signal became sparser and weaker following cytoplasmic disintegration (Fig. 7). In contrast, no fluorescence was observed in control groups (Fig. 8). Thus, US10 is primarily localized within the cytoplasm.

### US10 Protein is a component of the virion

**Fig. 9:**
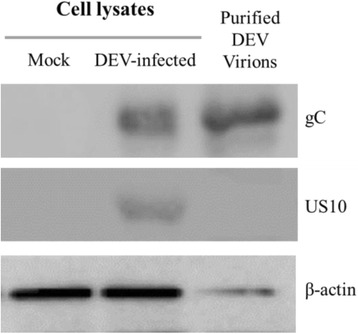
Western blot analysis of purified DEV virions. Virions purified from DEF cells were separated by SDS-PAGE, transferred to PVDF membranes, and probed with antibodies against the US10 protein and a control glycoprotein C envelope protein (gC). Total mock-infected or infected cell lysates were also included as an antibody control

The anti-US10 serum was used to probe the lysates of mock-infected cells, DEV-infected cells, and purified DEV virions (anti-gC serum was used as a positive control). The US10 protein could only be detected in DEV-infected cells, while gC protein could be detected in both purified virions and DEV-infected cells (Fig. 9).

The purified virions were also analyzed by mass spectrometry to identify the protein content. We identified 40 structural proteins in the purified DEV virions (data not shown), including DEV US10 protein (Table 2). One unique peptide of DEV US10 was detected, while three unique peptides matched DEV gC (*P* < 0.05). The relative abundance of US10 was low based on the exponentially modified protein abundance index (emPAI). From the results of the above two methods, DEV US10 is a minor component of the virion and the failure of western blotting may be caused by its low abundance.

**Table 2:** Viral content of DEV extracellular virions (partial)

Protein	Description	Score	Mass	Matches	Sequences	emPAI	NCBI Accession
UL44	glycoprotein C	97	47,836	6 (3)	6 (3)	0.22	AJG04885
US10	Virion protein	23	34,402	4 (1)	4 (1)	0.10	AGW24857

## Discussion

Herpesvirus genes are expressed sequentially in three distinctly defined stages: α (immediate-early), β (early), and γ (late) genes [1, 15]. The majority of the γ gene products are virion structural proteins that contribute primarily to virion assembly and morphogenesis. Our research demonstrated that the DEV US10 gene can be classified as a γ2 gene, as determined by western blotting, RT-PCR, and pharmacological inhibition experiments. The results are consistent with a report on the herpes simplex virus 1 (HSV-1) US10 gene [10], which was shown to encode a tegument protein. The function of HSV-1 US10 has not yet been characterized.

We also observed the subcellular localization of DEV US10 proteins by indirect immunofluorescence. They were abundantly distributed in the cytoplasm, specifically localizing around the nucleus, which was in accordance with earlier observations of Marek’s disease virus (MDV) US10 proteins [23]. MDV US10 has been reported to specifically bind to chicken stem cell antigen 2 (SCA2, a putative Marek’s disease resistance gene), but the mechanism and significance of their interaction are not well defined [12].

Recently, mass spectrometry has been widely used to analyze the structural proteome of herpesviruses, such as KSHV, HCMV, EBV, PRV, HSV-1, BoHV-4, and BoHV-1 [20–22, 24–28]. It has been proved that this approach is sufficiently sensitive to detect low-abundance proteins such as HSV-1 UL6 (a protein present in only 12 copies in mature virions), or the smallest proteins predicted to present in virions, such as HSV-1 US9 and UL11 (90 and 96 aa, respectively) [20]. To determine whether the DEV US10 protein is a structural component, mass spectrometry and western blotting were used to detect it in purified virions. Surprisingly, US10 was successfully detected by mass spectrometry, but not by western blotting. Similar conflicting result has also been reported on HSV-1 ICP0 [29]. This discrepancy may be a result of US10 being a low-copy virion protein, which is difficult to detect with antibody. Hence, we assume that DEV US10 protein is a minor component of extracellular virions.

Overall, our data suggest that US10 is a γ2 gene that probably functions as a virion protein, similar to HSV-1 US10 [10]. These data provide a foundation for future studies on duck enteritis virus US10.

## Conclusions

Through experiments involving qRT-PCR, western blotting, and pharmacological inhibition tests, the DEV US10 gene was identified as a true late (γ2) gene. In infected cells, DEV US10 protein was initially diffusely distributed throughout the cytoplasm and gradually relocated to a perinuclear region. DEV US10 is detected in mature virions, so it is a virion protein. However, further studies are still needed to explore its functions.

## Abbreviations

BoHV-1: Bovine herpesvirus type 1
BoHV-4: Bovine herpesvirus type 4
CHX: Cycloheximide
DAPI: 4′,6-diamidino-2-phenylindole
DEF: Duck embryo fibroblast
DEV: Duck enteritis virus
EBV: Epstein–Barr virus
EHV: Equine herpesvirus
GCV: Ganciclovir
HCMV: Human cytomegalovirus
HSV: Herpes simplex virus
IFA: Indirect immunofluorescence
IPTG: Isopropyl thiogalactoside
KSHV: Kaposi’s sarcoma-associated herpesvirus
LC-MS/MS: Liquid chromatography–tandem mass spectrometry
MDV: Marek’s disease virus
PRV: Pseudorabies virus
PVDF: Polyvinylidene fluoride
RT-PCR: Real-time quantitative reverse-transcription PCR
SDS-PAGE: Sodium dodecyl sulfate polyacrylamide gel electrophoresis
